# Avian Influenza A(H9N2) Virus Transmission across Chicken Production and Distribution Networks, Vietnam

**DOI:** 10.3201/eid3202.251416

**Published:** 2026-02

**Authors:** Mathew Hennessey, Thuy Hoang Thi, Jayna Raghwani, Younjung Kim, Hoa Thi Thanh Pham, Trang Huyen Nguyen, Hung Quoc Nguyen, Joshua G. Lynton-Jenkins, Ashley C. Banyard, Ian H. Brown, Joe James, Tom Lewis, Nicola S. Lewis, Dirk Pfeiffer, Fiona Tomley, Damer Blake, Ngoc Pham Thi, Anne Conan, Vuong Nghia Bui, Guillaume Fournié

**Affiliations:** Royal Veterinary College, London, UK (M. Hennessy, J. Raghwani, N.S. Lewis, D. Pfeiffer, F. Tomley, D. Blake, G. Fournié); National Institute of Veterinary Research, Hanoi, Vietnam (T.H. Thi, T.H. Nguyen, H.Q. Nguyen, N.P. Thi, V.N. Bui); University of Oxford, Oxford, UK (Y. Kim); Université de Montpellier, Montpellier, France (H.T.T. Pham, A. Conan); World Organisation for Animal Health/Food and Agriculture Organization International Reference Laboratory for Avian Influenza Animal and Plant Health Agency, Addlestone, UK (J.G. Lynton-Jenkins, A.C. Banyard, J. James, T. Lewis); Pirbright Institute, Pirbright, UK (I.H. Brown); World Health Organization Collaborating Centre for Influenza Reference and Research, London (N.S. Lewis); City University, Hong Kong, China (A. Conan); French Agricultural Research Centre for International Development, Harare, Zimbabwe (A. Conan); Université de Lyon, Marcy l’Etoile, France (G. Fournié); Université Clermont Auvergne, Saint Genes Champanelle, France (G. Fournié)

**Keywords:** influenza, avian influenza, H9N2, viruses, zoonoses, Vietnam, respiratory infections

## Abstract

In northern Vietnam, during March 2021–March 2022, prevalence of influenza A(H9N2) in chickens was higher in distribution facilities than on farms and varied between facility types. Phylogenetic analysis indicated extensive viral mixing along networks of chicken production and distribution, highlighting a need for risk mitigation across the entire network.

High-pathogenicity avian influenza A subtype H5N1 viruses of the Goose/Guangdong/1/96 lineage and low pathogenicity avian influenza A subtype H9N2 viruses are endemic in Southeast Asia, including Vietnam. Both subtypes affect poultry production and pose zoonotic risks (*1*) directly and through their involvement in the generation of novel virus reassortments (*2*). In Vietnam, the subtypes are frequently detected in live bird markets, and higher prevalence is associated with practices such as mixing poultry from multiple sources (*3*). However, that finding should be interpreted cautiously because farms and the markets they supply have limited prevalence data, and the focus on markets has diverted attention from larger slaughter facilities despite those facilities playing a key role in poultry distribution. Together, those gaps constrain understanding of avian influenza virus (AIV) transmission risk along the production and distribution network (PDN) through which poultry are raised, traded, and consumed. We conducted a cross-sectional study in northern Vietnam to assess avian influenza A(H5N1) and A(H9N2) prevalence in chickens, determine how AIV prevalence varied between farms and the different distribution facility types, and examine how viral genetic diversity was structured along the PDN.

## The Study

**Figure 1:**
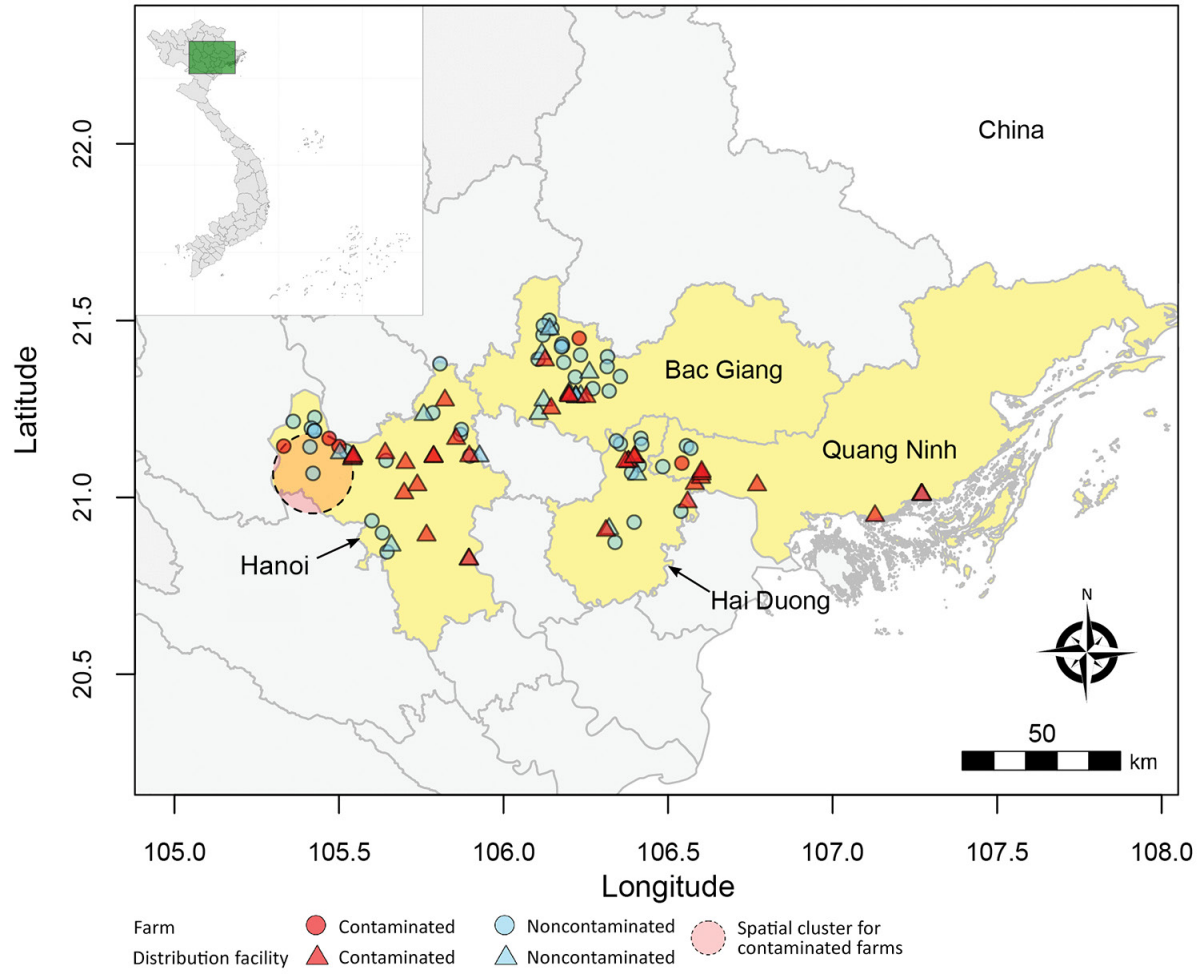
Geographic area and sites for study of avian influenza virus A(H9N2) prevalence across chicken production and distribution networks, Bac Giang, Hanoi, Hai Duong, and Quang Ninh Provinces, Vietnam, March 2021–March 2022. The 4 study provinces and the individual sites (farms and distribution facilities) are shown alongside the detection of avian influenza viruses (contaminated defined as >1 positive sample; noncontaminated defined as all samples testing negative) and the spatiotemporal cluster of contaminated farms. The 2 other farms that appear located in the cluster were not sampled during the temporal window.

We selected 50 farms and 52 distribution facilities, including retail and wholesale markets, small-scale slaughter points, and industrial slaughterhouses, across 4 provinces (Bac Giang, Ha Noi, Hai Duong, and Quang Ninh) (Figure 1). We identified distribution facilities trading or processing slow-growing colored broiler chickens (i.e., hybrids of indigenous roosters and fast-growing broiler hens that account for most chicken meat produced in Vietnam [*4*]) through consultation with provincial authorities and traders. We randomly selected 4 retail markets and 4–5 slaughter points in the urban area of each province and recruited all wholesale markets and slaughterhouses in the study area. We traced farming areas supplying distribution facilities through interviews and snowball sampling. We randomly selected 1 farm per distribution facility from the list of farms of these supplying areas. During March 2021–March 2022, we collected oropharyngeal and cloacal swab specimens from 15 slow-growing broilers at each site and from fast-growing broilers when present. On farms, we sampled flocks near the end of production cycles. We administered structured questionnaires to capture husbandry, vaccination, and trade practices. Our study was approved by the National Institute of Veterinary Research (Vietnam) (approval no. 020-480433/DD-YTCC) and the Royal Veterinary College (UK) Ethics and Welfare Committee (approval no. URN: 20204811983-3). Participants were informed about the study and then asked to provide their verbal consent to take part.

We screened samples for the influenza A virus matrix gene by using real-time reverse transcription PCR (*5*) and then subtyped positive samples (i.e., those with cycle threshold values <40) for H5 and H9 (Appendix 1). We sequenced samples with cycle threshold values <30, compared them with genomes from Vietnam during 2018–2022 for which sequences were available through GISAID (https://www.gisaid.org), and submitted the genomes to GISAID (Appendix 2). We conducted phylogenetic analyses by using Bayesian methods to infer lineage structure and reassortment patterns (*6*). We performed separate analysis for distribution facilities and farms by using space-time cluster analysis by using a Bernoulli model (SaTScan, https://www.satscan.org) and Bayesian hierarchical logistic regression models (https://doi.org/10.32614/CRAN.package.R2jags) to estimate AIV prevalence at site and bird levels.

All farms were independently owned and held 100–15,000 (median 2,000) slow-growing broilers 70–240 days old (mean 116 days) at sampling. Sixty percent of farmers reported AIV vaccination for their flocks.

Among 1,682 sampled chickens (750 from farms and 932 from distribution facilities), a single chicken from a Quang Ninh slaughter point tested positive for H5, a finding consistent with farm-level H5N1 virus prevalences of 0%–0.1% reported in Egypt and Bangladesh (*7*,*8*). Depending on their pathogenicity, some H5N1 virus lineages can circulate in waterfowl without causing overt clinical disease. Spillover into chickens has frequently been associated with close proximity to free-grazing ducks and rice paddy ecosystems (*9*), and proximity of these species within markets probably facilitates cross-species transmission. In contrast, we detected H9N2 virus in 11.7% (197/1,682) of all sampled chickens. On farms, estimated bird-level prevalence of H9N2 virus was 4.5%, and detection was more frequent in unvaccinated (4/20) than vaccinated (1/30) flocks, although that difference was not significant (p = 0.14 by Fisher exact test). Such high prevalence in farmed chickens is similar to that for Egypt but higher than for previous reports from southern Vietnam and Bangladesh (*7*,*10*,*11*).

The best-fitting model differentiated distribution facilities by type and supplying area (Appendix 1 Table 4), whereas including chicken type or sampling period did not improve the model fit. Overall, bird-level prevalence in distribution facilities was ≈5-fold higher than on farms and was highest in informal slaughter points, followed by retail markets, slaughterhouses, and wholesale markets (Table). Influenza A(H9N2) virus prevalence was nearly as high on farms as in wholesale markets and was estimated to increase by 254% (95% highest density interval 4%–754%) from farms to retail markets and 545% (95% highest density interval 108%–1396%) from farms to slaughter points (Table).

**Table:** Posterior estimates and posterior predictive values of avian influenza virus A(H9N2) prevalence across chicken production and distribution networks, Bac Giang, Ha Noi, Hai Duong, and Quang Ninh Provinces, Vietnam, March 2021–March 2022*

Site	No. sites	No. chickens	H9N2-positive chickens, no (%)	H9N2 prevalence, median % (95% HDI)		
				Site-level	Bird-level in contaminated sites	Overall† bird-level
Farms	50	750	31 (4.1)	0.111 (0.037–0.202)	0.414 (0.305–0.525)	0.045 (0.014–0.086)
Distribution facilities	52	932	168 (18.0)			
Retail market	16	240	38 (15.8)	0.778 (0.559–0.967)	0.212 (0.123–0.309)	0.161 (0.088–0.244)
Wholesale market	11	210	14 (6.7)	0.481 (0.163–0.850)	0.125 (0.033–0.238)	0.057 (0.014–0.117)
Slaughter point	19	363	106 (29.2)	0.725 (0.527–0.898)	0.413 (0.308–0.515)	0.295 (0.190–0.402)
Slaughterhouse	6	119	10 (8.4)	0.233 (0.014–0.542)	0.483 (0.158–0.795)	0.103 (0.002–0.286)

*HDI, high density interval. †Estimated prevalence across both contaminated and uncontaminated sites.

**Figure 2:**
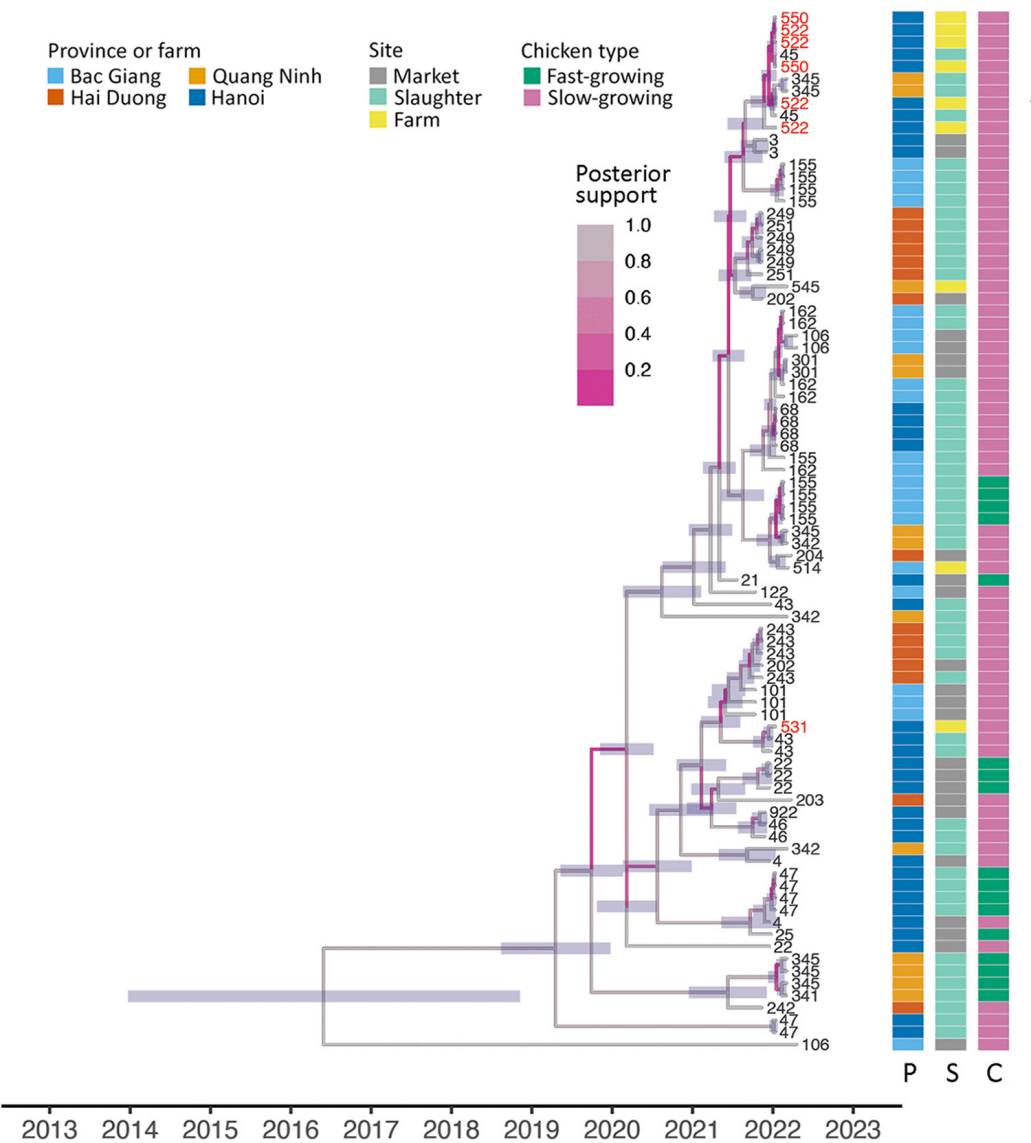
Time-scaled phylogenies of hemagglutinin sampled avian influenza virus A(H9N2) virus genomes in study of H9N2 virus prevalence across chicken production and distribution networks, Bac Giang, Hanoi, Hai Duong, and Quang Ninh Provinces, Vietnam, March 2021–March 2022. Tree tips are labeled by the unique sampled site identification; red text indicates sequences from 3 farms in the spatiotemporal cluster. Branches are colored by posterior support, and horizontal node bars represent 95% highest posterior density intervals of node ages. Heatmaps indicate province, site type, and chicken type of each sequence. C, chicken type; P, province; S, site type.

**Figure 3:**
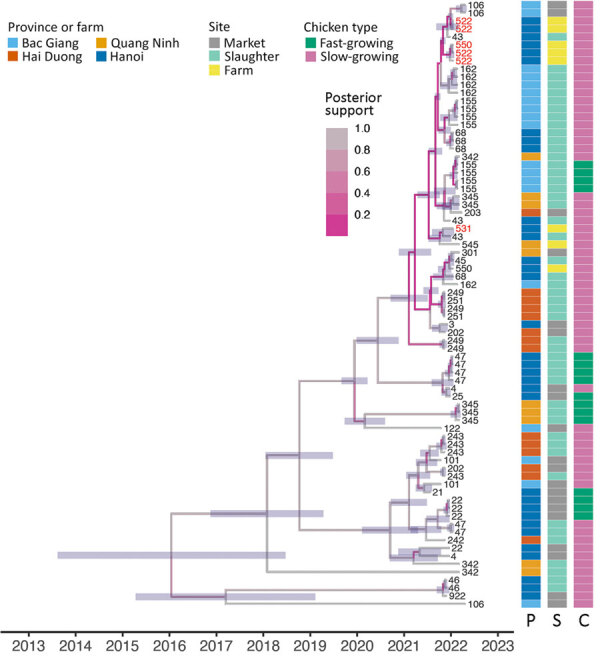
Time-scaled phylogenies of neuraminidase of sampled avian influenza virus A(H9N2) virus genomes in study of H9N2 virus prevalence across chicken production and distribution networks, Bac Giang, Hanoi, Hai Duong, and Quang Ninh Provinces, Vietnam, March 2021–March 2022. Tree tips are labeled by the unique sampled site identification; red text indicates sequences from 3 farms in the spatiotemporal cluster. Branches are colored by posterior support, and horizontal node bars represent 95% highest posterior density intervals of node ages. Heatmaps indicate province, site type, and chicken type of each sequence. C, chicken type; P, province; S site type.

On the basis of H9 sequences, we assigned all viruses to clade B4.71 (maximum identity >96%). Time-scaled phylogenies of H9N2 virus genomes showed limited genetic diversity (Figure 2, 3; Appendix 1 Figure 5, 6). The estimated time to most recent common ancestor for all sampled viruses spanned a broad range (95% highest posterior density interval 2008–2018 across all segments), although most sequences shared a more recent time to most recent common ancestor (95% highest posterior density interval ≈2017–2019). Our dataset included sequences from 3 farms forming a spatiotemporal cluster in Hanoi (23.5-fold higher risk than for farms outside; p = 0.045) (Figure 2, 3; Appendix 1 Figure 5, 6). Viruses from 2 farms had high genetic identity, consistent with epidemiologic linkage, whereas virus from a third farm (site 531) clustered separately in all 8 gene segments, suggesting that the spatiotemporal cluster involved >2 genetically distinct lineages. Alongside detection of genetically distinct viruses within and between distribution facilities, these findings demonstrate the presence of distinct H9N2 virus lineages at local scales and frequent reassortment in the region (Figure 2, 3).

## Conclusions

Our results indicate that poultry production and distribution network configurations, including the origins of supplying farms, influence H9N2 virus prevalence and thus human exposure risk. The pattern of viral circulation amplification along the PDN is consistent with studies in southern Vietnam and Bangladesh (*7*,*11*) and probably reflects trading conditions. For example, birds are mixed from multiple sources and experience lengthy, stressful transportation (i.e., crowding and lack of sustenance), creating opportunities for virus exposure and transmission en route; that finding was supported by the improved model fit when we included supplying areas, suggesting that procurement and distribution practices influence viral prevalence at distribution facilities. 

High viral prevalence in slaughter points and retail markets probably reflects sourcing involving multiple suppliers, including wholesale markets, indicating that chickens spent longer in the PDN before sampling. The lack of geographic structure in AIV genetic diversity further points to extensive viral mixing among poultry populations. Control and surveillance efforts should therefore address the entire poultry PDN. 

The Vietnam Avian and Human Influenza Preparedness program has prioritized upgrading wholesale markets and industrial slaughterhouses and promoting their use (*12*). Although low prevalence in such facilities supports this focus, slaughterhouses remain underused, and informal slaughter points are still preferred (*13*). Surveillance and risk mitigation strategies must also target the numerous distribution facilities in Vietnam that are small and informal but widely used.

Appendix 1 Additional information about methods and results in study of avian influenza A(H9N2) virus transmission across chicken production and distribution networks, Vietnam.

Appendix 2 Additional information about sample genomes sequenced in study of avian influenza A(H9N2) virus transmission across chicken production and distribution networks, Vietnam.
