# Supplementary material for: Avian Influenza A(H9N2) Virus Transmission across Chicken Production and Distribution Networks, Vietnam
Source: Emerg Infect Dis. 2026 Feb;32(2):259–64. doi: 10.3201/eid3202.251416 (PMC12928235; doi:10.3201/eid3202.251416)
Supplement: Appendix 1 — Additional information about methods and results in study of avian influenza A(H9N2) virus transmission across chicken production and distribution networks, Vietnam. [file 25-1416-Techapp-s1.pdf]

Article DOI: https://doi.org/10.3201/eid3202.251416

EID cannot ensure accessibility for supplementary materials supplied by authors. Readers who have difficulty accessing supplementary content should contact the authors for assistance.

Avian Influenza A(H9N2) Virus Transmission across Chicken Production and Distribution Networks, Vietnam

Appendix 1

Methods

Site eligibility criteria

Between March 2021 and March 2022, we visited sites in four provinces of northern Viet Nam (Bac Giang, Ha Noi, Hai Duong, and Quang Ninh) representing areas with dense poultry (i.e., chickens and ducks) and human populations.

Distribution facilities that were active during the study period and traded or processed slow-growing broiler chickens were classified into markets (retail and wholesale) and slaughter facilities (slaughter points and industrial slaughterhouses). A market was an open space with at least two live chicken vendors operating at least once a week and where other goods may also be traded (*1*). Wholesale markets in Viet Nam are typically large, with over 20 vendors, selling chickens to traders operating in other locations. Retail markets are generally smaller with all chickens directly sold to consumers, restaurateurs, or caterers. Industrial slaughterhouses process large numbers of chickens using semi-automatised systems, while slaughter points handle smaller amounts (tens to hundreds of birds per day) manually. As there was only a small number of wholesale markets and industrial slaughterhouses in the study provinces, all such sites were selected. They were identified through consultation with the Department of Animal Health (DAH) and the Food and Agriculture Organization of the United Nations (FAO), and interviews with poultry traders during field investigations.

Retail markets and slaughter points in urban areas (i.e., wards in cities and towns) of the four provinces were eligible whereas rural sites were excluded. In Ha Noi, where live bird

marketing was prohibited by the Ha Noi People's Committee (Decisions 71/2007/ QĐ-UBND) in the urban center wards, surrounding districts were included. Four to five wards were randomly selected per province, with one site for each distribution facility randomly chosen per ward.

For each selected distribution facility, the farming areas supplying them with slow-growing broiler chickens were identified through interviews with vendors and mobile traders, tracing the supply chain back to the production sites at the sub-district (commune) level. For each distribution facility, one commune within the identified supply area was randomly selected, all its eligible farms were listed with the assistance of local DAH, and one was randomly selected. Farms holding ≥100 slow-growing broiler chickens at a late production stage (i.e., >70 days-old) were eligible. At each site, cloacal and oropharyngeal swabs were collected from 15 slow-growing broiler chickens to ensure ≥95% confidence in detecting AIV at a prevalence of ≥20%. If fast-growing broiler chickens were present, up to 15 were also sampled.

Structured questionnaires were administered to owners of farms and distribution facilities to collect data on poultry husbandry, including flock size, vaccination, age at sampling, numbers sold in markets or processed in slaughter facilities. Questionnaires were developed in English by UK and Vietnamese partners, translated into Vietnamese, and piloted before revision. Responses were entered using the Vietnamese version of the survey tool ODK and translated into English for analysis by Vietnamese partners.

Sample screening

Samples were screened by the National Institute of Veterinary Research (NIVR), Viet Nam, for the influenza A virus matrix gene using real-time reverse transcription polymerase chain reaction (rRT-PCR) (*2*). Virus RNA (vRNA) was extracted using the QIAamp Viral RNA kit (Qiagen, Germany) following manufacturer's instructions (*3*). Samples with a cycle threshold (C_t) <40 were transferred to the Animal and Plant Health Agency (APHA), UK, for H9 and H5 subtyping by RT-PCR (*4*,*5*). A sample was positive if C_t <36 (*6*), and a bird was positive if either oropharyngeal or cloacal swab was positive. Samples with C_t≤30 were considered for sequencing, with two to six randomly selected per site and chicken type, proportional to prevalence.

Sequencing and bioinformatics

Whole genome sequence (WGS) data was generated at APHA following previously described methods (*7*). Influenza virus genomes were assembled from raw sequence data with the IRMA pipeline (*8*), which resulted in between 76–89 sequences with >75% sequence coverage per gene segment. These sequences were combined with publicly available H9N2 virus sequences sampled between 2018 and 2022 from GISAID. HA sequences were classified using the "A/H9 influenza virus lineage and clade assignment" tool (*9*).

Phylodynamic analysis

Complete H9N2 virus sequences were retrieved for all eight gene segments identified in Viet Nam from 2018 to 2023 via GISAID (Appendix 2, https://wwwnc.cdc.gov/EID/article/32/2/25-1416-App2.xlsx). These sequences were combined with newly generated data from this study for each gene segment and aligned using MAFFT v7.487 (*10*). The evolutionary history of each gene segment was reconstructed using a Bayesian phylogenetic framework implemented in BEAST v1.10.4 (*11*). Specifically, we applied a codon-structured substitution model, a Bayesian skyline coalescent prior, and a relaxed log-normal distributed molecular clock. For each gene segment, two independent Markov chain Monte Carlo (MCMC) runs of 50 million steps were performed, with sampling conducted at regular intervals to obtain 10,000 samples per chain. Maximum clade credibility trees were visualized with R packages 'ggtree' and 'treeio' (*12*). Subtrees containing virus sequences generated from this study were extracted using the *drop.tip* function in 'treeio' package.

Space-time cluster analysis

We assessed whether contaminated distribution facilities and farms clustered over space or both space and time. A contaminated site had ≥1 AIV-positive chicken. A Bernoulli model using 999 permutations was specified using SaTScan (*13*). Both spatial circular and elliptic windows were used, with a maximum size of 50% of the population at risk. Temporal precision was 1 month, with clusters ranging from 1 month to 50% of the study period. Significance was set at $p < 0.05$.

Prevalence estimation

Bayesian zero-inflated logistic regression models were built to estimate H9N2 prevalence at site and bird levels, separately for distribution facilities and farms. For distribution facilities,

site- and bird-level factors that may influence prevalence were considered (*1*): facility type (*2*), chicken type (slow-, fast-growing broiler) (*3*), facility province or (*4*) supplying area, and (*5*) sampling period, with January–February (Têt festival) defined as high-risk period (*14*). Wholesale markets and slaughterhouses being unevenly distributed, distribution facility- and session-related variables were included only in models differentiating distribution facility types, and separately due to limited sample size (Table 2). Parameters were estimated by MCMC in R2JAGS (*15*) in R (*16*), and compared using deviance information criterion (DIC), with differences ≥5 indicating better fit [32,33].

Model specification

Prevalence estimation

Separate bayesian zero-inflated logistic regression models were constructed for distribution facilities and farms with the following structure. The number Y_{si} of chickens of type i testing positive in site s follows a binomial distribution,

$$Y_{si} \sim Binomial(\theta_{si}, n_{si})$$

With n_{si} the number of chickens of type i sampled in site s and θ_{si} the chicken-level prevalence in site s:

$$\theta_{si} = C_s \delta_{si}$$

$$C_s \sim Bernoulli(\omega_t)$$

With C_s the contamination status of site s. It follows a Bernoulli distribution, with ω_t the probability of a site of type t being contaminated. Thus, in a contaminated site, $\theta_{si} = \delta_{si}$, the probability of a chicken testing positive. δ_{si} is formulated as a logistic regression

$$logit(\delta_{si}) = \alpha_s + \sum_j \beta_j A_{sij}$$

β_j is a regression coefficient associated with a chicken- or site-level characteristic j, A_{sij} an indicator variable, equal to 1 if chickens of type i in site s, or the site s, shows this characteristic j, and null otherwise. α_s is a site-specific random intercept, assumed to follow a normal distribution with mean μ and variance σ^2:

$$\alpha_s \sim Normal(\mu, \sigma^2)$$

Under the null model, chickens and sites were not differentiated based on their characteristics, with $\forall t\ \omega_t = \omega$ and $\forall i, t\ logit(\delta_{si}) = \alpha_s$. Due to the limited number of positive chickens in farms, only a null model without random effect was considered to estimate the prevalence of H9N2 in these settings. δ_{si} could then be reformulated as $\delta_{si} = \mu$. On the other hand, multiple models with different sets of chicken- or site-level variables were considered when estimating the prevalence of H9N2 in distribution facilities, as mentioned in the manuscript. The types of distribution facility were either differentiated as (i) markets and slaughter facilities, or (ii) retail markets, wholesale markets, slaughter points and slaughterhouses. The distribution facilities' locations were Ha Noi, Bac Giang or Hai Duong/Quang Ninh as most sites sampled in Hai Duong and Quang Ninh were near their shared border, and the distribution facilities' supplying areas were Ha Noi, Bac Giang, Hai Duong/Quang Ninh or Other provinces.

Weakly informative priors were specified, $Normal(0,100)$ for the regression coefficients and the mean of the random intercept, $InverseGamma(1,1)$ for the variance of the random intercept, $Beta(1,1)$ for the probability of a site being contaminated and the probability of a chicken testing positive in the farm model. After a burn-in of 10,000 iterations, models were considered to have achieved convergence based on the visual inspection of traceplots and if Gelman statistics were <1.001 and effective sample size was >10,000 for each parameter (*17*). As a posterior predictive check, the contamination status of each site and the infection status of each sampled chicken in contaminated site were simulated, and summary statistics were compared to the observations. The median and highest density interval (HDI) of each parameter's marginal posterior distribution were computed as well as odds ratios where appropriate. For models with distribution facilities differentiated according to their type and location or supplying area, the overall viral prevalence for each distribution facility type was averaged as follows. First, parameter values were sampled from the joint posterior distribution. The posterior predicted prevalence in each sampled distribution facility was obtained by multiplying the corresponding estimated site-level and chicken-level prevalence. These posterior predicted prevalence estimates were then averaged with respect to the distribution facility type and province. This was repeated for 10,000 iterations, and medians and HDI computed for the resulting distributions.

Model script

```
model
{
for(i in 1:no_site_by_type) {
positive[i] ~ dbin(p_bird.1[i], total[i])
p_bird.1[i] <- i_endpoint[site_id[i]]*p_bird[i]
p_bird[i] <- ilogit(intercept_bird +
p_bird_bird_type[i] +
p_bird_type[site_id[i]] +
p_bird_prov[site_id[i]] +
random_site[site_id[i]])
p_bird_bird_type[i] <- 0
}
for(j in 1:no_site) {
i_endpoint[j] ~ dbern(p_endpoint[site_type[j]]) # uses 'endpoint', 'site_type', or 'sales'
p_bird_type[j] <- β_T1*retail[j] + β_T2*wholesale[j] + β_T3*shouse[j]
p_bird_prov[j] <- β_P1*cHN[j] + β_P2*cBG[j] + β_P3*cHD_QN[j] + β_P4*cOT[j]
random_site[j] ~ dnorm(0, 1/sigma_1^2)
}
# β priors for endpoint
p_endpoint[1] <- p_endpoint_1
p_endpoint[2] <- p_endpoint_2
p_endpoint[3] <- p_endpoint_3
p_endpoint[4] <- p_endpoint_4
```

```
p_endpoint_1 ~ dbeta (1,1)
p_endpoint_2 ~ dbeta (1,1)
p_endpoint_3 ~ dbeta (1,1)
p_endpoint_4 ~ dbeta (1,1)
intercept_bird ~ dnorm(0, 1/SD^2)
β_T1 ~ dnorm(0, 1/SD^2)
β_T2 ~ dnorm(0, 1/SD^2)
β_T3 ~ dnorm(0, 1/SD^2)
β_P1 ~ dnorm(0, 1/SD^2)
β_P2 ~ dnorm(0, 1/SD^2)
β_P3 ~ dnorm(0, 1/SD^2)
β_P4 ~ dnorm(0, 1/SD^2)
sigma_1 <- 1 / sqrt(tau)
tau ~ dgamma (1,1)
}
```

Additional results

Farm practices

Of the 52 tested farms, all but two raised only chickens; two farms also raised ducks and were negative for AIV.

Most farmers (30/50, 60%) reported AIV vaccination, but only three specified the subtype, all H5N1. Vaccination occurred at a median age of 28 days (range 10–50) with a median 87 days (40–165) between vaccination and sampling.

Chickens sold through distribution facilities

Wholesale markets sold far more chickens daily (median: 2906, range: 239–115,050) than retail markets (93, 11–759), and slaughterhouses (550, 143–2250) processed more than

slaughter points (25, 4–125). Overall, fast-growing broiler chickens were sampled in seven (36.8%) slaughter points, three (50%) slaughterhouses, three (27.3%) wholesale markets and no retail markets.

Farms supplying distribution facilities were located across the four study provinces and others in northern Viet Nam. Most distribution facilities (46/52, 88.5%) were supplied, at least partially, by farms in their own province (Appendix 1 Table 1). About half (27/52, 51.9%) were supplied by one province, this was highest for slaughter points (63.2%) and lowest for wholesale markets (33.3%), the latter also most often supplied by non-study provinces (72.7%).

Across farms and distribution facilities, 213 chickens (12.6%) were M gene-positive, 197 (11.7%) H9-positive, and one H5-positive – a slow-growing broiler from a Quang Ninh slaughter point. All but one H9-positive chickens had oropharyngeal positivity, 6 had cloacal positivity (Appendix 1 Table 2). Subsequent WGS confirmed all H9- and H5-positive samples as H9N2 and H5N1, respectively.

A total of 750 slow-growing broilers were sampled from 50 farms. Across 52 distribution facilities, 932 chickens were sampled (748 slow-growing, 184 fast-growing), from four provinces (Appendix 1 Table 3).

AIV modeling

All models converged (Appendix 1 Figure 1,2).

For distribution facilities, the best-fitting model differentiated sites by facility type (retail market, wholesale market, slaughter point, slaughterhouse) and by the location of the supplying farms (Ha Noi, Bac Giang, Hai Duong/Quang Ninh) and had a DIC 20.2 units lower than the null model (Appendix 1 Table 4). This model also outperformed a comparable model which used facility location instead of supplying farm location, with a DIC difference of 5.3 units. One additional model within 5 DIC units of the best-fitting model included chicken type (Slow-growing broiler, fast-growing broiler); however, estimates of shared coefficients were similar across models.

Simulations from the best-fitting model reproduced positive chicken counts, overall and by distribution facility type, though prevalence was overestimated in Bac Giang, Ha Noi and Quang Ninh (Appendix 1 Figure 3).

Marginal posterior distributions for the best fitting distribution facility model are shown in Appendix 1 Table 5, Figure 4.

Phylodynamic and spatiotemporal cluster analysis

Sequences from the three farms (sites 522, 531, 550) in the Ha Noi spatiotemporal cluster highlighted contrasted patterns: viruses from two farms (522, 550) located around 4km from each other (Appendix 1 Table 6) clustered together, while another (531) located further away in the cluster consistently grouped separately across all segments (Appendix 1 Figure 5,6).

For context, in HA, this corresponded to four amino acid and eight nucleotide differences between farm 531 and the two others; in PB2, five amino acid and 16 nt differences. Therefore, the spatiotemporal farm cluster comprised at least two distinct viral lineages. Additionally, TMRCA of these sites varied across segments, from 0.77 years in NA to 2.18 years in HA. These observations together with frequent detection of genetic diversity within and between distribution facilities support co-circulation of divergent lineages at local scales and frequent reassortment in the region.

References

1. Fournié G, Guitian J, Desvaux S, Mangtani P, Ly S, Cong VC, et al. Identifying live bird markets with the potential to act as reservoirs of avian influenza A (H5N1) virus: a survey in northern Viet Nam and Cambodia. PLoS One. 2012;7:e37986. PubMed https://doi.org/10.1371/journal.pone.0037986

2. Centers for Disease Control and Prevention. CDC real-time RT-PCR (rRT-PCR) protocol for detection and characterization of swine influenza (version 2009). Reference. no. I-007–05. 2009 [cited 2025 Jun 4]. https://www.hsdl.org/c/view?docid=778749

3. QIAGEN. QIAamp Viral RNA Mini handbook. 2023 [cited 2024 Dec 9]. https://www.qiagen.com/us/resources/resourcedetail?id=c80685c0-4103-49ea-aa72-8989420e3018&lang=en

4. Slomka MJ, Hanna A, Mahmood S, Govil J, Krill D, Manvell RJ, et al. Phylogenetic and molecular characteristics of Eurasian H9 avian influenza viruses and their detection by two different H9-specific realtime reverse transcriptase polymerase chain reaction tests. Vet Microbiol. 2013;162:530–42. PubMed https://doi.org/10.1016/j.vetmic.2012.11.013

5. Slomka MJ, Pavlidis T, Banks J, Shell W, McNally A, Essen S, et al. Validated H5 Eurasian real-time reverse transcriptase-polymerase chain reaction and its application in H5N1 outbreaks in 2005-2006. Avian Dis. 2007;51(Suppl):373–7. PubMed https://doi.org/10.1637/7664-060906R1.1

6. Animal and Plant Health Agency. Detection of influenza A matrix gene by real-time Taqman RT-PCR SOP VI.493. 2020 [cited 2023 Oct 18]. https://science.vla.gov.uk/fluglobalnet/Documents/english/protocol_Taqman.pdf

7. Byrne AMP, James J, Mollett BC, Meyer SM, Lewis T, Czepiel M, et al. Investigating the genetic diversity of H5 avian influenza viruses in the United Kingdom from 2020–2022. Microbiol Spectr. 2023;11:e0477622. PubMed https://doi.org/10.1128/spectrum.04776-22

8. Shepard SS, Meno S, Bahl J, Wilson MM, Barnes J, Neuhaus E. Viral deep sequencing needs an adaptive approach: IRMA, the iterative refinement meta-assembler. BMC Genomics. 2016;17:708. PubMed https://doi.org/10.1186/s12864-016-3030-6

9. Fusaro A, Pu J, Zhou Y, Lu L, Tassoni L, Lan Y, et al.; International H9 Evolution Consortium. Proposal for a global classification and nomenclature system for A/H9 influenza viruses. Emerg Infect Dis. 2024;30:1–13. PubMed https://doi.org/10.3201/eid3008.231176

10. Katoh K, Standley DM. MAFFT multiple sequence alignment software version 7: improvements in performance and usability. Mol Biol Evol. 2013;30:772–80. PubMed https://doi.org/10.1093/molbev/mst010

11. Suchard MA, Lemey P, Baele G, Ayres DL, Drummond AJ, Rambaut A. Bayesian phylogenetic and phylodynamic data integration using BEAST 1.10. Virus Evol. 2018;4:vey016. PubMed https://doi.org/10.1093/ve/vey016

12. Yu G. Using ggtree to visualize data on tree-like structures. Curr Protoc Bioinformatics. 2020;69:e96. PubMed https://doi.org/10.1002/cpbi.96

13. Kulldorff M. SaTScan. 2005 [cited 2023 Oct 18]. https://www.satscan.org

14. Delabouglise A, Choisy M, Phan TD, Antoine-Moussiaux N, Peyre M, Vu TD, et al. Economic factors influencing zoonotic disease dynamics: demand for poultry meat and seasonal transmission of avian influenza in Vietnam. Sci Rep. 2017;7:5905. PubMed https://doi.org/10.1038/s41598-017-06244-6

15. Su YS, Yajima M. R2jags. 2021 [cited 2023 Jun 8]. https://cran.r-project.org/web/packages/R2jags/R2jags.pdf

16. R Core Team. R: a language and environment for statistical computing. 2022 [cited 2023 Oct 10]. https://www.r-project.org

17. Gelman A, Carlin JB, Stern HS, Dunson DB, Vehtari A, Rubin DB. Bayesian data analysis. Third edition. Boca Raton (Florida): Taylor and Francis; 2014. Chapter 11, p. 281–6.

Appendix 1 Table 1. The distribution of distribution facilities according to their location, type and supplying areas

		Supplying areas				Location			
Distribution facilities	*n*	Ha Noi	Bac Giang	Hai Duong/ Quang Ninh	Non-study provinces	Ha Noi	Bac Giang	Hai Duong	Quang Ninh
Type									
Retail market	16	6	7	7	4	4	4	4	4
Wholesale market	11	9	5	2	8	7	3	1	0
Slaughter point	19	8	7	8	5	5	5	5	4
Slaughterhouse	6	3	2	0	4	3	3	0	0
Location									
Ha Noi	19	19	2	1	12				
Bac Giang	15	1	14	0	4				
Hai Duong	10	3	1	9	5				
Quang Ninh	8	3	4	7	0				

Appendix 1 Table 2. Details of AIV screening

		H9N2		
Gene		Positive	Negative	Total
M gene	Positive	195	16*	211
	Negative	2	0	2
	Total	197	16	213

*One sample from a slow-growing broiler from a slaughter point in Quang Ninh was positive for H5N1.

Appendix 1 Table 3. Recruited sites and H9N2 prevalence. H9N2+ (%): number and proportion of sites with ≥1 positive chicken or proportion of positive chickens

	Sites		All chickens		Slow-growing		Fast-growing	
Site	n	H9N2+ (%)	n	H9N2+ (%)	n	H9N2+ (%)	n	H9N2+ (%)
Farms	50	5 (10%)	750	31 (4.1%)	750	31 (4.1%)	-	-
Distribution facilities	52	31 (59.6%)	932	168 (18.0%)	748	130 (17.4%)	184	38 (20.7%)
Retail market	16	12 (75.0%)	240	38 (15.8%)	240	38 (15.8%)	-	-
Wholesale market	11	4 (36.4%)	210	14 (6.7%)	165	6 (3.6%)	45	8 (17.8%)
Slaughter points	19	14 (73.7%)	363	106 (29.2%)	269	76 (28.3%)	94	30 (31.9%)
Slaughterhouse	6	1 (16.7%)	119	10 (8.4%)	74	10 (13.5%)	45	0 (0%)

Appendix 1 Table 4. Distribution facility models*

Distribution facility type	Bird type	Location	Supplying area	Season	DIC	∆DIC
		null model			220.4	20.2
	C				222.1	21.9
A					221.5	21.3
A	C				223.3	23.1
B					215.8	15.6
B	C				217.2	17.0
B		D			205.5	5.3
B	C	D			207.8	7.6
**B**			**E**		**200.2**	**0**
B	C		E		202.1	1.9
B				F	215.7	15.5
B	C			F	217.6	17.4

*Best-fitting model in bold type. A- Market, slaughter facilities. B- Retail market, wholesale market, slaughter point, slaughterhouse. C- Slow-growing broiler, fast-growing broiler. D- Ha Noi, Bag Giang, Hai Duong/Quang Ninh (most Hai Duong/Quang Ninh sites were near their shared border). E- Ha Noi, Bag Giang, Hai Duong/Quang Ninh, Other provinces. F- High-risk season, low-risk season

Appendix 1 Table 5. Model estimations. H9N2 distribution facility model posterior estimates

Parameter	Posterior values	Odds Ratio (95% HDI)
Site-level		
Distribution facility type		
Retail market	0.778 (0.559,0.967)	
Wholesale market	0.481 (0.163,0.850)	
Slaughter point	0.725 (0.527,0.898)	
Slaughterhouse	0.233 (0.014,0.542)	
Bird-level		
Intercept	0.511 (−0.428,1.446)	
Distribution facility type		
Slaughter point	Reference	1
Retail market	−1.122 (−1.893,-0.362)	0.33 (0.12,0.62)
Wholesale market	−1.544 (−2.792,-0.38)	0.21 (0.03,0.56)
Slaughterhouse	−0.059 (−1.851,1.705)	0.94 (0.01,3.94)
Supplying area		
Ha Noi	−1.661 (−2.546,-0.764)	0.19 (0.06,0.41)
Bac Giang	−0.006 (−0.876,0.867)	0.99 (0.3,2.09)
Hai Duong/Quang Ninh	−0.787 (−1.614,0.043)	0.46 (0.15,0.93)
Other provinces	0.460 (−0.332,1.237)	1.58 0.58,3.13)

Appendix 1 Table 6. Summary of approximate distances in kilometers between the A(H9N2) positive farms in the spatiotemporal cluster

Farm	522	531	550
522	-	22km	4km
531	22km	-	19km
550	4km	19km	-

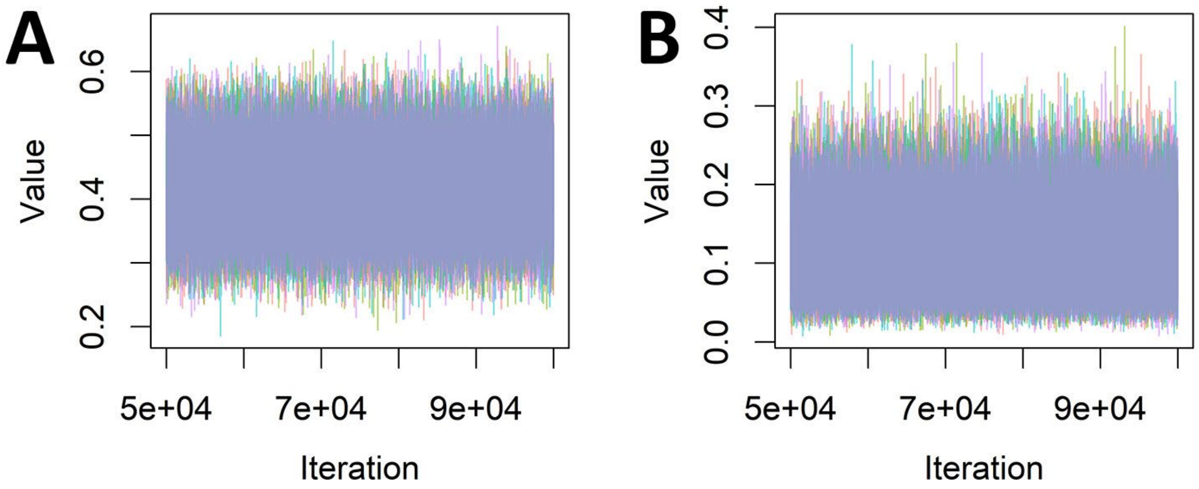

Appendix 1 Figure 1. Traceplots for farm model.

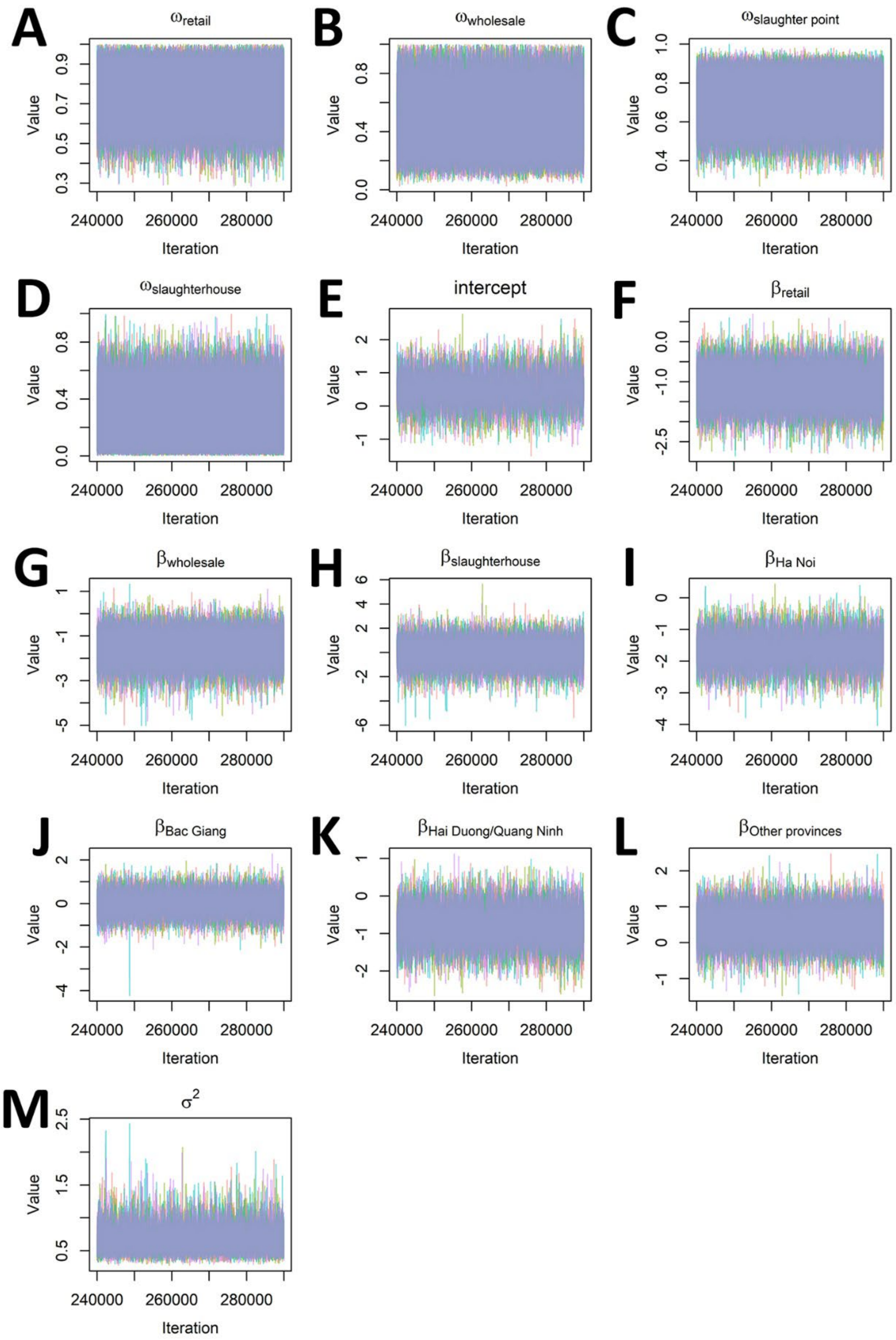

Appendix 1 Figure 2. Traceplots for best fitting distribution facility model.

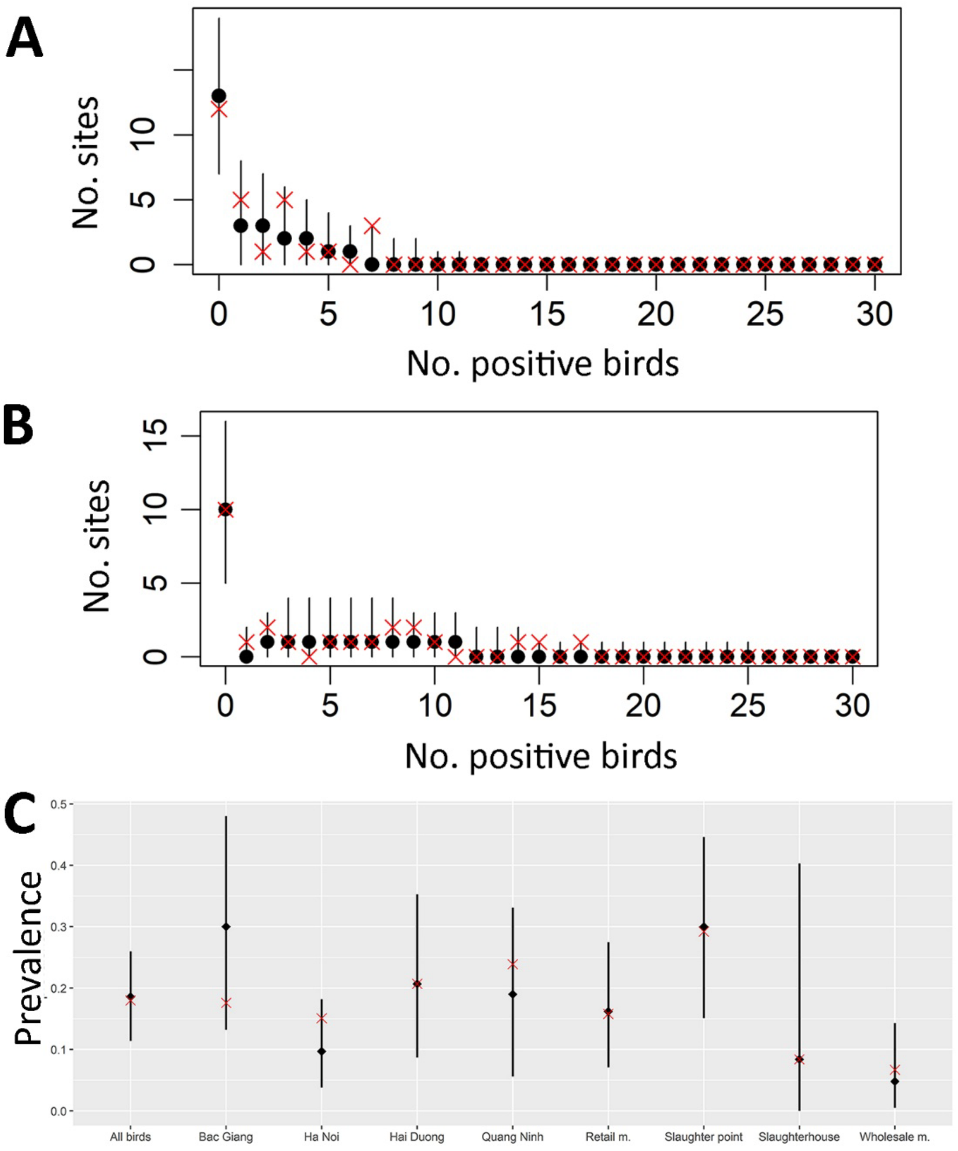

Appendix 1 Figure 3. Post-predictive checks for the best fitting distribution facility model. Distribution of the number of market (A) and slaughter (B) distribution facilities according to the number of birds that tested positive for H9N2 in the site. Simulated data represented in black; median number of sites (black dot), 95% HDI (black line) and empirical data from study (red cross). Proportion of birds that tested positive for H9N2 (C); overall, in each province, and in each site type; simulated median prevalence (black dot), 95% HDI (black line) and empirical data from study (red cross).

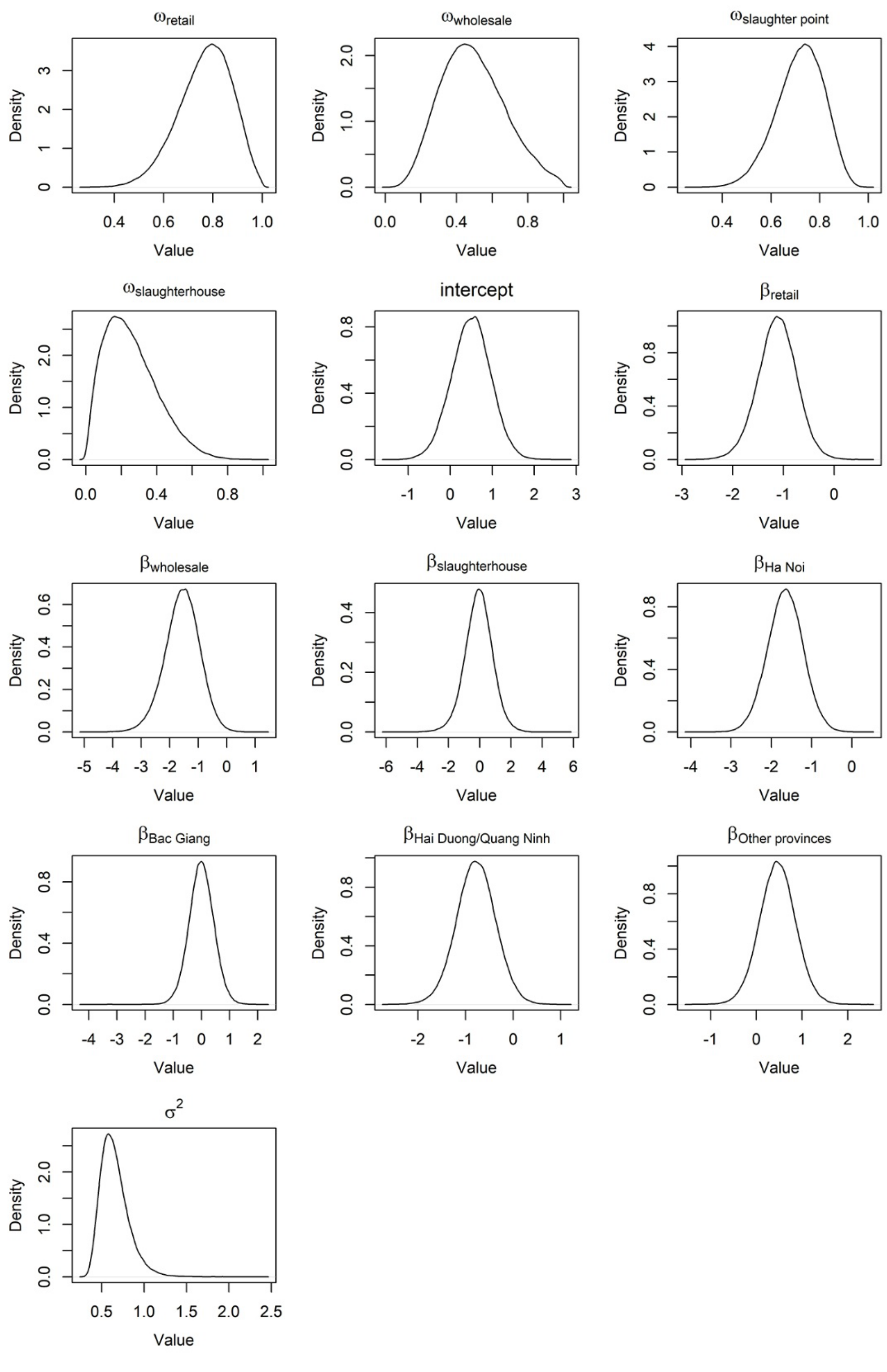

Appendix 1 Figure 4. Density plots of marginal posterior distributions of the best fitting distribution facility model parameters.

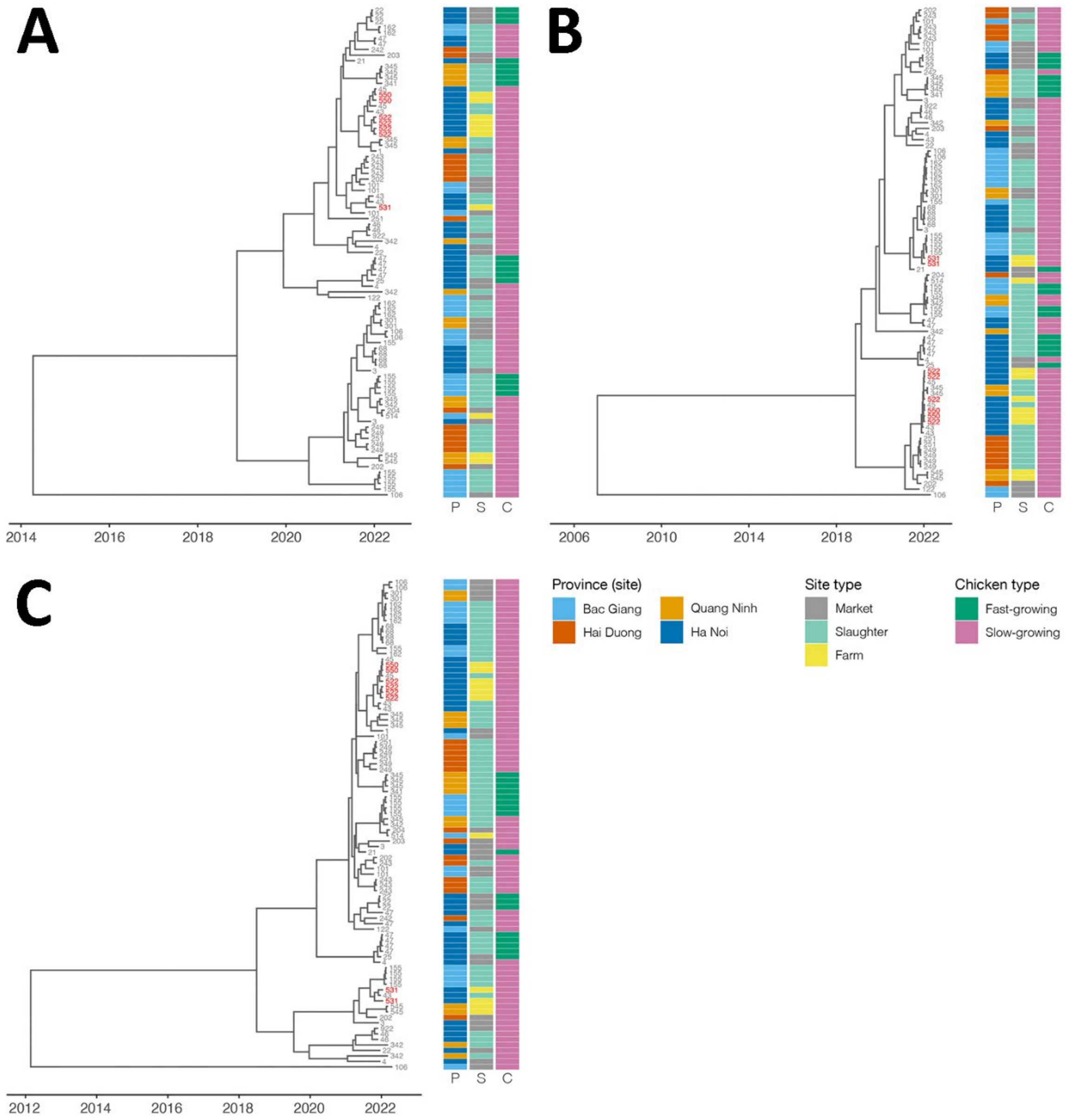

Appendix 1 Figure 5. Time-scaled phylogenies of PB2 (A), PB1 (B), and PA (C) of sampled H9N2 virus genomes. Tips are labeled by the unique sampled site ID. Province, site type, and chicken type of each sequence are indicated by three heatmaps. Sequences from three farms in the spatiotemporal cluster are highlighted in red.

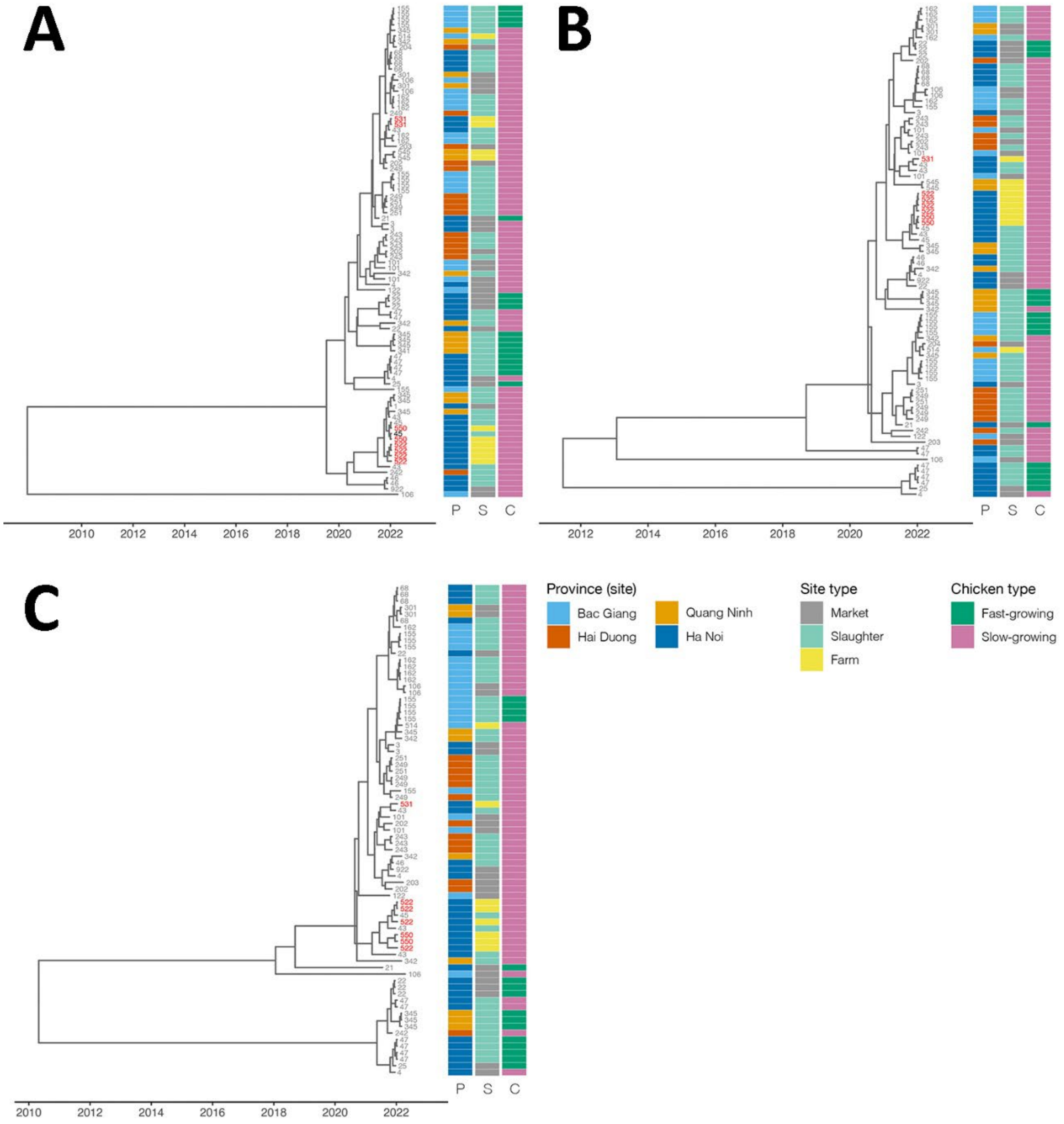

Appendix 1 Figure 6. Time-scaled phylogenies of NP (A), MP (B), and NS (C) of H9N2 virus genomes sampled from this study. Tips are labeled by the unique ID of each sampled site. Province, site type, and chicken type of each virus sequence are indicated by three heatmaps. Sites highlighted in red corresponds to virus sequences from three farms associated with spatiotemporal cluster.
